# Machine learning for estimation and control of quantum systems

**DOI:** 10.1093/nsr/nwaf269

**Published:** 2025-07-07

**Authors:** Hailan Ma, Bo Qi, Ian R Petersen, Re-Bing Wu, Herschel Rabitz, Daoyi Dong

**Affiliations:** School of Engineering, Australian National University, Canberra, ACT 2601, Australia; Nanyang Quantum Hub, School of Physical and Mathematical Sciences, Nanyang Technological University, Singapore 637371, Singapore; State Key Laboratory of Mathematical Sciences, Academy of Mathematics and Systems Science, Chinese Academy of Sciences, Beijing 100190, China; School of Mathematical Sciences, University of Chinese Academy of Sciences, Beijing 100049, China; School of Engineering, Australian National University, Canberra, ACT 2601, Australia; Center for Intelligent and Networked Systems, Department of Automation, Tsinghua University, Beijing 100084, China; Department of Chemistry, Princeton University, Princeton, NJ 08544, USA; Australian Artificial Intelligence Institute, Faculty of Engineering and Information Technology, University of Technology Sydney, Sydney, NSW 2007, Australia

**Keywords:** quantum estimation, quantum control, quantum measurement, machine learning, neural network, reinforcement learning

## Abstract

The advancement of quantum technologies depends on the ability to create and manipulate increasingly complex quantum systems, with critical applications in quantum computation, quantum simulation and quantum sensing. These developments present substantial challenges in efficient control, calibration and verification of quantum systems. Machine learning methods have emerged as powerful tools owing to their remarkable capability to learn from data, and have thus been extensively utilized for various quantum tasks. This paper reviews several significant topics at the intersection of machine learning and quantum estimation and control. Specifically, we discuss neural network–based approaches for quantum state estimation, gradient-based methods for quantum optimal control, evolutionary computation for learning control of quantum systems, machine learning techniques for quantum robust control and reinforcement learning for adaptive quantum control.

## Teaser

This article reviews cutting-edge machine learning approaches driving advances in quantum system estimation and control for next-generation quantum technologies.

## INTRODUCTION

Estimation and control of quantum systems are fundamental in advancing quantum technologies, experiencing notable progress over the past three decades; for an overview, see, e.g. the survey papers [1–5] and monographs [6–8]. Acquiring information about unknown quantum entities can be realized by performing measurements on quantum systems and deducing patterns from measured results. Owing to the considerable ability of machine learning (ML) to extract useful patterns in large-scale and complex data, it is highly desirable to apply ML to assist in the post-processing of measurement data. Quantum control, on the other hand, focuses on directing the evolution of quantum systems with the objective often being to maximize a specific performance function [2]. ML offers distinct advantages in searching for control policies without knowing the exact model of quantum systems [5]. In this review, we present a comprehensive introduction to both quantum estimation and quantum control tasks, highlighting the integration of ML techniques within these domains [9].

Rooted in the foundational principles of pattern recognition and statistical learning theory, ML has evolved to encompass a broad spectrum of learning paradigms [10], including learning from data, e.g. supervised learning (data classification) and unsupervised learning (data clustering), or learning from interaction, e.g. reinforcement learning (decision making). One attractive model in ML is neural networks (NNs), where researchers have found that multiple, sequential, hidden feedforward layers have additional benefits [11]. For example, convolutional NNs (CNNs), known for their translation invariance, have achieved success in the fields of vision and pattern recognition [12]. Recurrent neural networks (RNNs) have been proposed for dealing with sequential data or time series data, such as long short-term memory (LSTM) [13]. Transformers employ an attention mechanism to capture long-range dependencies more effectively [14], thereby achieving great success in natural language processing and computer vision [15]. In the field of generative models, generative adversarial networks (GANs) [16] and variational autoencoders (VAs) [17] are two successful approaches. Diffusion models operate by first transforming images into Gaussian distributions through a forward diffusion process, and then iteratively sampling the new images from this noisy state using a reverse denoising process, showcasing exceptional capabilities in image synthesis [18]. Flow matching builds upon continuous normalizing flows, offering an efficient framework for generative tasks with improved training stability and sampling efficiency [19]. Another type of ML is reinforcement learning (RL) (see [20] for a review), which was initially developed for robotics, but has been extensively applied to other fields that involve sequential decision-making processes (e.g. AlphaGo [21]).

Recently, quantum-mechanical formalism has been incorporated into ML, known as quantum machine learning, and has demonstrated ‘quantum advantage’ in sample complexity or time complexity when dealing with some learning and optimization problems. Quantum neural networks have achieved great success owing to their expressivity and generalization (see [22] for a review). Through deliberate designs using quantum circuits, these components can be leveraged for tasks such as estimating wave functions [23] and reconstructing unitary operations [24]. Researchers have found that quantum machines can learn from exponentially fewer experiments compared to conventional methods. This exponential advantage has been demonstrated in tasks such as predicting properties of physical systems, performing quantum principal component analysis and learning physical dynamics [25]. Additionally, quantum speedup has been observed in quantum RL [26], where this learning protocol is implemented on a compact and fully tunable integrated nanophotonic processor [27]. There are already several comprehensive review papers about quantum machine learning (see, e.g. [28,29]). In this paper, we mainly focus on machine learning methods for quantum applications.

It is a fundamental task to characterize the state or the evolution of a quantum system. This typically involves reconstructing full or partial characteristics from measured statistics, collectively referred to as learning a complex distribution. ML provides a data-driven technique to extract useful patterns from data, which suggests a natural benefit of robustness against noise in measurement data [30,31]. Within the framework of function approximation by learning from labeled data, NNs have been widely investigated for quantum state tomography (QST) [32–35]. Among them, different architectures have been applied, with the Transformer architecture being used to capture long correlations among constituent qubits, i.e. quantum entanglement [36]. Compared to conventional methods, NNs aim to capture key patterns by approximating a complex function from large-scale data. This ability brings robustness against possible noise in the data, making NNs promising for reconstructing quantum states from imperfect measurement data [30,37]. Drawing from classical autoencoders [17], quantum autoencoders have been proposed to reorganize high-dimensional states into latent representations that can be potentially recovered with high fidelity, thus saving valuable resources [38–40]. Additionally, quantum metrology studies the estimation of the parameters of quantum systems, which relies on identifying optimal probe states, evolution processes and measurement operators [41]. ML methods offer a distinct solution to adaptive learning of quantum systems. For example, an adaptive Bayesian approach updated the evolution time, contributing to the efficient use of resources (i.e. the number of experiments) for phase estimation [42].

Another significant task in quantum technology is the design of a target quantum evolution, which can be tackled by quantum control. Its goal is to identify how the control fields of physical systems can be adapted to achieve the desired evolution [1]. This underlying problem often manifests as an optimization problem under realistic constraints, posing challenges for conventional optimizers. Learning-based control approaches have been developed for the manipulation of various quantum systems [2], where different learning algorithms (e.g. greedy algorithms [43,44] or global approaches [45,46]) iteratively suggest improved control fields based on prior trial experiments [2,47]. By incorporating the concept of sampled-based learning, the optimized control pulses exhibit robustness against uncertain parameters in system Hamiltonians [47]. Complementary to learning-based optimization, identifying optimal strategies can also be realized with real-time feedback from quantum systems [48,49]. This constitutes an active learning process where an RL *agent* is designed to learn a policy rather than the optimization of a particular control field [50–52]. This model-free approach allows for more autonomy and flexibility (i.e. the same machinery can be used in additional settings without alteration). Incorporating NNs into RL not only enables flexible representations of a *state* (e.g. wave function, density matrix) and an *action* (e.g. discrete or continuous controls), but also makes it possible to learn a robust control policy by learning from large-scale data [50,53]. Flexible representation using NNs accommodates the inherent properties of quantum stochasticity and partial observability. This is significant for quantum experiments when only partial observations of quantum systems are available (see, e.g. [51,53,54]). Deep reinforcement learning (DRL) methods have been extensively applied to quantum error correction [48,51,54] and other applications (see [55,56] for quantum compiling, and [57–59] for quantum metrology).

In this review, we attempt to provide a selected overview of ML’s applications in quantum technologies. Specifically, we delve into quantum estimation challenges by leveraging data-driven learning techniques and address the complexities involved in controlling quantum systems by utilizing ML methods. The remainder of this paper is organized as follows. We first provide background information on quantum estimation, quantum control and several fundamental concepts in quantum mechanics. Then, we investigate the integration of ML in quantum estimation tasks and the performance of learning-based optimization of quantum systems. The utilization of RL for quantum control is discussed, followed by an outlook.

## PRELIMINARIES

We first briefly introduce several related concepts for estimation and control of quantum systems, including quantum states, quantum measurements and quantum evolution. Then, we introduce several concepts related to ML methods.

### Fundamental concepts in quantum mechanics

#### Quantum state

In quantum mechanics, the Dirac notation $|\psi \rangle$ is commonly used to represent a pure state of a finite-dimensional closed quantum system. Mathematically, $|\psi \rangle$ corresponds to a unit vector in a complex Hilbert space $\mathbb {H}$ and is referred to as a wave function. Quantum information can be encoded using two-level quantum systems, known as qubits, whose general state can be expressed as


(1) \begin{equation*}
|\psi \rangle = a_0 |0\rangle + a_1 |1\rangle ,
\end{equation*}

where $a_0, a_1 \in \mathbb {C}$ and $|a_0|^2 + |a_1|^2 = 1$. Here, $|0\rangle$ and $|1\rangle$ correspond to the classical bit values 0 and 1, respectively [6]. Since the global phase of a quantum state has no physical observable consequences, states $|\psi \rangle$ and $e^{\mathrm{i}\phi }|\psi \rangle$ (with $\mathrm{i} = \sqrt{-1}$ and $\phi \in \mathbb {R}$) are considered physically indistinguishable.

For open quantum systems, their states cannot be written in the form of unit vectors as Equation (1). In such cases, the density operator $\rho$ is introduced to describe the state of a quantum system. Let $(\cdot )^{\dagger }$ denote the adjoint operation, and we have $\langle \psi _j| =(|\psi _j\rangle )^{\dagger }$. A density operator can be represented as an ensemble of pure states $\lbrace |\psi _j\rangle \rbrace$, i.e.


(2) \begin{equation*}
\rho = \sum _j p_j |\psi _j\rangle \langle \psi _j|,
\end{equation*}

where $p_j \ge 0$ and $\sum _j p_j=1$. Hence, a density operator $\rho$ is Hermitian, and positive semi-definite, and has unit trace, i.e. it satisfies $\rho =\rho ^{\dagger }$, $ \rho \ge 0$ and $\mbox{Tr}(\rho )=1$. In the special case of a pure state $|\psi \rangle$, the density matrix reduces to $\rho = |\psi \rangle \langle \psi |$, and satisfies $\mbox{Tr}(\rho ^2) = 1$.

#### Quantum measurement

In the fields of quantum control and quantum engineering, extracting information from quantum systems is a fundamental task. Unlike classical physics, quantum measurement theory introduces unique challenges, as a measurement performed on a quantum system usually disturbs the system itself. A comprehensive discussion of this phenomenon can be found in [60]. A quantum measurement is associated with a collection $\lbrace \mathcal {M}_j\rbrace$ of measurement operators, acting on the state space of the system being measured and satisfying the completeness equation


(3) \begin{equation*}
\sum _j \mathcal {M}_j^{\dagger } \mathcal {M}_j = \mathcal {I},
\end{equation*}

where $ \mathcal {I}$ denotes the identity matrix and $j$ labels the possible measurement outcomes. For a quantum system in state $|\psi \rangle$, the probability that the $j$th result occurs is given by


(4) \begin{equation*}
p_j = \langle \psi | \mathcal {M}_j^{\dagger } \mathcal {M}_j |\psi \rangle .
\end{equation*}

Upon obtaining outcome $j$, the state of the measured system collapses to $\mathcal {M}_j |\psi \rangle / \sqrt{p_j}$. The completeness condition ensures that the total probability is normalized, i.e. $\sum _j p_j = 1$.

A POVM (Positive Operator-Valued Measure) generalizes projective measurements. Specifically, a set of operators $E_j$ is known as the POVM elements associated with the measurement, and the corresponding probability is given by $p_j=\langle \psi | E_j | \psi \rangle$. A widely used measurement model is the projective measurement, satisfying $\mathcal {M}_j=\mathcal {M}_j^{\dagger }, \mathcal {M}_{j}\mathcal {M}_{j^{\prime }}= \delta _{jj^{\prime }} \mathcal {M}_j$, where $\delta _{jj^{\prime }}$ represents the Kronecker delta. Define projectors as $\mathcal {P}_j = \mathcal {M}_j^{\dagger } \mathcal {M}_j$ and the probability of the $j$-th outcome is given as $p_j = \langle \psi | \mathcal {P}_j |\psi \rangle$.

#### Quantum evolution

The evolution of a closed quantum system is governed by a unitary transformation. State $ |\psi \rangle$ of the system at time $ t_1$ evolves to state $ |\psi ^{\prime }\rangle$ at time $ t_2$ according to $|\psi ^{\prime }\rangle = \mathcal {U} |\psi \rangle$, with $\mathcal {U} \mathcal {U}^{\dagger }= \mathcal {I}$. For mixed states, we have $\rho ^{\prime } = \mathcal {U} \rho \mathcal {U}^{\dagger }$. Quantum gates can be expressed as unitary operators. For example, the *Hadamard* gate has the corresponding unitary matrix


\begin{eqnarray*}
\frac{1}{\sqrt{2}}
\left [\begin{array}{cc}
1 &\quad \ 1 \\
\ 1 &\quad -1
\end{array}\right].
\end{eqnarray*}

If the quantum system under consideration has interaction with its environment, it becomes an open quantum system and has a more complicated (usually non-unitary) evolution. To deal with this situation, quantum processes are proposed to describe the time evolution of an (open) quantum system (also known as a quantum dynamical map), which is a linear map from the set of density matrices to itself. Let $\Lambda$ be a map that transforms an input state $\rho _{\text{in}}$ into an output state


(5) \begin{equation*}
\rho _{\text{out}} = \Lambda (\rho _{\text{in}}).
\end{equation*}

For a physical quantum map, $\Lambda$ must be completely positive.

According to the Choi–Jamiolkowski isomorphism [61], there exists a one-to-one correspondence between every quantum map $\Lambda$ and a Choi operator $Q_{\,\text{Choi}}$, such that


(6) \begin{equation*}
\Lambda (\rho _{\text{in}})=\mbox{Tr}_A[Q_{\,\text{Choi}}(\rho _{\text{in}}^\top \otimes \mathcal {I})],
\end{equation*}

where $\mbox{Tr}_A(\rho )$ denotes the partial trace corresponding to subsystem $A$ [6], and we have


(7) \begin{equation*}
Q_{\,\text{Choi}}=\sum _{ij} |i\rangle \langle j| \otimes \Lambda (|i\rangle \langle j|),
\end{equation*}

indicating that $Q_{\,\text{Choi}}$ characterizes $\Lambda$ completely.

### Estimation of quantum systems

In numerous applications across quantum information and quantum engineering, it is essential to acquire information about an unknown quantum system; i.e. to identify structural features or estimate relevant parameters. This highlights the significance of quantum estimation, commonly referred to as quantum tomography (QT) [6,62]. Unlike its classical counterpart, QT typically operates under the assumption that a large number of independent, identical copies of an unknown quantum state are available. Information is then extracted by performing quantum measurements on these copies following certain protocols. To uniquely determine a quantum state, a set of informationally complete (or overcomplete) measurements is performed, with measured statistics given as $\boldsymbol{f}=[f_1,f_2,\dots ]^\top$, with $\sum _i f_i = 1$.

QT relies on the measured frequency vector $\boldsymbol{f}=[f_1,f_2,\dots ]^\top$ that is a statistical approximation to the true probability vector $\boldsymbol{p}=[p_1,p_2,\dots ]^\top$ (see Equation (4) for more details), in order to infer underlying information about quantum entities. Such a task can be summarized as obtaining an estimate of the entire entity (called full QT) or of partial properties of the entity. Following this framework, the estimation of quantum states is realized by determining the density matrices of a fixed state of quantum systems, while the estimation of the quantum process is realized by determining the evolution of quantum systems.

### Quantum control systems

The dynamics of a closed quantum system can be described by the Schrödinger equation:


(8) \begin{equation*}
\frac{\mathrm{d}}{\mathrm{d}t}|{\psi }(t)\rangle =-\frac{\rm {i}}{\hbar }\mathcal {H}(t) |{\psi (t)}\rangle
\end{equation*}

with $\hbar$ the Planck constant. Throughout this work, we adopt atomic units and set $\hbar =1$. Here $\mathcal {H}(t)$ is a Hermitian operator known as the Hamiltonian of the quantum system. Alternatively, the evolution can be described in terms of the density matrix via the Liouville–von Neumann equation:


(9) \begin{equation*}
\dot{\rho }(t)=-\mathrm{i}[\mathcal {H}(t), \rho (t)]
\end{equation*}

with $[A,B]=AB-BA$ denoting the commutator. For a quantum control system, the total Hamiltonian can be expressed as


(10) \begin{equation*}
\mathcal {H}(t)=\mathcal {H}_0+\sum _{m=1}^{N_c}u_m(t)\mathcal {H}_m,
\end{equation*}

where $\mathcal {H}_0$ is the time-independent free Hamiltonian, and the $\mathcal {H}_m$ are control Hamiltonians coupled to external fields $u_m(t)$. The unitary evolution $\mathcal {U}(t,t_0)$ from time $t_0$ to $t$ under the Hamiltonian can be given as


(11) \begin{equation*}
\mathcal {U}(t,t_0)=\mathcal {T}_{\leftarrow } \bigg [\exp \bigg (-{\rm {i}}\int _{t_0}^{t}\mathcal {H}(t^{\prime })dt^{\prime }\bigg )\bigg ],
\end{equation*}

where $\mathcal {T}_{\leftarrow }$ represents time ordering [60]. Quantum control aims at searching for a set of control fields $\lbrace u_m(t)\rbrace$ to drive the quantum system to achieve a given target with the desired performance.

When a quantum system interacts with its environment (i.e. a dissipative bath coupled to a quantum system), the system becomes an open one and its dynamics under Markovian approximation can be described by the Markovian master equation (MME) [1]:


(12) \begin{equation*}
\dot{\rho }(t)=-{\rm {i}}[\mathcal {H}(t),\rho (t)]+\sum _k \eta _k \mathcal {D}[L_k](\rho (t)).
\end{equation*}

Here $\mathcal {D}[L_k](\rho )=L_k \rho L_k^{\dagger }-\frac{1}{2} L_k^{\dagger } L_k \rho -\frac{1}{2}\rho L_k^{\dagger } L_k$, where $\lbrace L_k\rbrace$ are the operators coupling with the environment and the coefficients $\eta _k\ge 0$ characterize the relaxation rates.

In feedback control, continuous monitoring of the system is often used to acquire real-time information for feedback [63]. The evolution of a quantum system under continuous homodyne measurements of a field observable coupled with the system through an operator $L$ can be described by the stochastic master equation (SME)


(13) \begin{eqnarray*}
\dot{\rho }(t)\! &=&\! -\mathrm{i}[\mathcal {H}(t), \rho (t)] + \kappa \mathcal {D}[L](\rho (t))\nonumber\\
&&+\, \sqrt{\kappa } {\mathcal {H}}[L](\rho (t)) \mathrm{d} W_t,\nonumber\\
\mathcal {H}[L](\rho (t))\! &=&\! L\rho (t)+\rho (t)L^{\dagger }\!-\! \langle L+L^{\dagger }\rangle \rho (t),\nonumber\\
\end{eqnarray*}

where $\kappa \in (0,1]$ quantifies the measurement strength and $\langle \cdot \rangle =\mbox{Tr}(\cdot \rho )$ denotes the quantum expectation value. The term $\mathrm{d} W_t$ represents a Wiener increment with zero mean and variance $\mathrm{d}t$ and is related to the measurement output $y_t$ through the equation


(14) \begin{equation*}
\mathrm{d} y_t=\mathrm{d} W_t+ \sqrt{\kappa } \langle L+L^{\dagger } \rangle \mathrm{d} t.
\end{equation*}

It is worth noting that Equation (13) represents only one typical form of the SME; various other forms exist, each corresponding to a specific type of measurement process[64].

### Machine learning methods

ML focuses on the development of statistical algorithms that enable systems to learn from data and generalize to previously unseen situations. We refer to such a model as an *agent*. A key component of ML is access to large datasets or the ability to generate data synthetically. The training strategy for the *agent* varies depending on the task and typically falls into several distinct categories.

• *Supervised learning.* The training samples are labeled with their target values. The *agent* learns to map inputs to these predefined outputs.
• *Unsupervised learning*. The data come without labels, and the *agent* learns to uncover hidden patterns among the data.
• *Reinforcement learning.* Unlike the above paradigms, no training data are required. Instead, the *agent* interacts with an *environment*, which is distinguished from the environment (or a dissipative bath that is coupled to a quantum system) in quantum contexts. The *agent* receives feedback in the form of *reward*, with the purpose of maximizing long-term performance.

ML techniques can be applied to a wide range of tasks, which are generally categorized into distinct types. Common examples include classification, where data points are assigned to predefined categories; regression, which involves learning a continuous function that maps input vectors to output values; and generative modeling, which aims to sample new data vectors that follow a similar distribution to the observed data.

The basic building block of modern ML architectures can be expressed as an artificial neuron. Its basic units are single-output nonlinear functions: $y=g(W x+b)$ with $g:\mathbb {R}^n \rightarrow \mathbb {R}$ a nonlinear activation function. Specifically, the weights $W$ and optional biases $b$ are trainable parameters that can be optimized during the learning process. Since a single neuron cannot capture complex relationships in the data, multiple neurons are organized into layers and interconnected to form a multilayer NN. Generally, NNs with at least a single hidden layer can approximate arbitrary functions (the NNs are usually very wide for complex functions), which forms the theoretical basis for using them in approximating relationships between different types of data [11]. A fully connected multilayer NN is called a multilayer perceptron (MLP). Different from MLPs that utilize fixed activation functions (i.e. fixed form of $g$) on nodes (‘neurons’), Kolmogorov–Arnold networks, featuring learnable activation functions, have emerged as a promising tool in the ML community [65]. To train NNs, one needs to choose a problem-specific cost function (e.g. a mean squared error for regression problems or a cross-entropy loss for classification problems) that may be minimized via stochastic gradient descent. A central challenge of ML algorithms is generalizability: the ability to perform well not only on the training data, but also on unseen (testing) data. NNs, when sufficiently large, are known to be universal function approximators [11]. However, their sizes need to be chosen with care: overly large networks can become difficult to train and may exhibit poor generalization due to overfitting, a phenomenon where the model memorizes the training data instead of learning underlying patterns. Interestingly, as model complexity increases, performance may initially deteriorate before improving again—a behavior known as the double-descent phenomenon [66].

In the RL paradigm, the interaction of the *agent* with its *environment* is usually described within the framework of Markov decision processes (MDPs), defined by a five-tuple $\langle \mathbb {S},\mathbb {A},\mathbb {P},\mathbb {R},\gamma \rangle$ [20]. Specifically, let $\mathbb {S}$ denote the set of *states*, which is distinguished from the state in quantum contexts; let $\mathbb {A}$ be the set of *actions* that can be taken; $\mathbb {P}$ represents the *state* transition probability; $\mathbb {R}$ represents the *reward* and $\gamma \in [0,1]$ is the discount factor. A policy $\pi$ maps the state space $\mathbb {S}$ to the *action* space $\mathbb {A}$, i.e. $\pi :\mathbb {S} \rightarrow \mathbb {A}$. The goal of RL is to find an optimal *action*  $a_t^{*}$ for each state $s_{t}$ that maximizes the cumulative discounted *reward*: $G_t = \sum _{k=0}^{T-t}\gamma ^{k}r_{t+k}$. To this end, the *reward* signal is designed by a human supervisor to evaluate the quality of the resulting *state* after an *action* is applied. Notably, it is possible to define a *reward* structure aligned with the desired goal without knowing the optimal *action*, which is a major distinction between RL and supervised learning. In the RL community, the *agent* interacts with its *environment* whose *state* can be either fully or only partially observed through a corresponding observation obtained after executing an *action* according to an underlying policy $\pi$. In this case, partially observable MDPs are proposed, where the observation is dependent on the current *state* and the previous *actions* [67]. RL methods can be classified into three categories (see Table 1): (i) value-based methods that first approximate value functions, e.g. $Q(s, a)$ represents the expected cumulative *reward* after taking *action*  $a$ in *state*  $s$ [68]) and then obtaining a policy, e.g. $a^{*}=\max _{a \in \mathbb {A}} Q(s,a)$; (ii) policy-based methods that directly approximate a policy function $a=\pi (s)$ [69]; (iii) actor-critic methods that combine value approximation and policy approximation. Notably, by approximating the value function or policy function using multilayer NNs, deep RL methods represent a step toward building autonomous systems that can accept raw data from the real world [70], without relying on (manually) designed feature vectors.

**Table 1.** A taxonomy of RL. Different methods can be classified into (i) value-based methods that optimize value functions; (ii) policy-based methods that optimize policy functions and (iii) actor-critic methods that jointly optimize value functions and policy functions.


(i) Value-based algorithms	$Q$ -learning [68]
	SARSA [71]
	Deep $Q$ network (DQN) [70]
(ii) Policy-based algorithms	Policy gradient [69]
	Trust region policy optimization [72]
	Proximal policy optimization (PPO) [73]
(iii) Actor-critic algorithms	Asynchronous advantage actor-critic (A3C) [74]
(learn policy and value	Deep deterministic policy gradient (DDPG) [75]
functions jointly)	Twin-delayed DDPG (TD3) [76]

## MACHINE LEARNING FOR QUANTUM ESTIMATION

Quantum estimation usually involves the reconstruction of full or partial characteristics from measured statistics, whose performance may be limited by the state-preparation-and-measurement (SPAM) errors. ML provides a means of building noise resilience into the post-processing of measurement data and, thus can be useful to assist in quantum estimation tasks. In the following, we first outline the process of converting quantum estimation into an inversion problem. Subsequently, we focus on QST and investigate the performance of machine learning for quantum estimation. Then, we present results on the estimation of quantum dynamics. This section concludes with a discussion of the outlook and open questions of ML-aided quantum estimation.

### Quantum estimation as an inversion task

**Figure 1.**
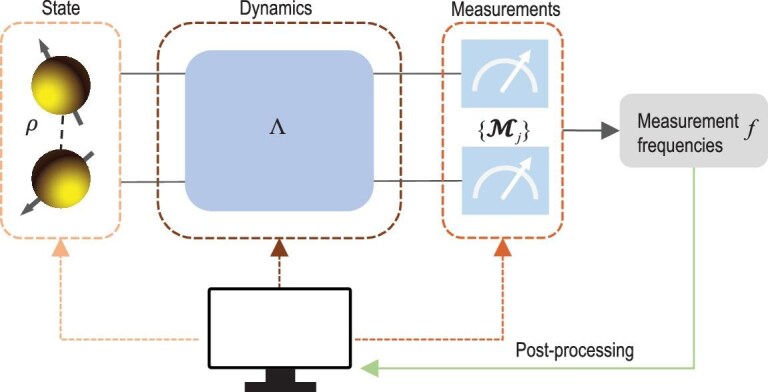
Schematic of quantum estimation, including estimating quantum states, dynamics and measurements. An initial quantum state $\rho$ undergoes a quantum operation $\Lambda$, ending up with an output state. Measurement frequencies $\boldsymbol{f}$ are collected by performing quantum measurements $\lbrace \mathcal {M}_j\rbrace$ on the output state. Quantum estimation aims to capture the underlying pattern among the observed data, which can be individually applied to deduce the parameters for the quantum state $\rho$, quantum evolution $\Lambda$ and quantum measurements $\lbrace \mathcal {M}_j\rbrace$, respectively.

Before introducing different solutions to quantum estimation, we first provide a broad overview of learning approaches for quantum systems at different levels involving quantum states, quantum dynamics and quantum measurements using post-processing techniques (see Fig. 1). Although the characterization of quantum systems can be realized using traditional methods (such as linear regression estimation [77], maximum likelihood estimation [78] and Bayesian estimation [79]), their solutions usually rely on informationally complete measurements or a large number of measurement copies. The complexity of quantum systems scales exponentially with their size, but in many practical scenarios, certain assumptions like low rank, sparsity or specific dynamics make it possible for classical algorithms to efficiently characterize unknown quantum entities [5].

For quantum estimation, the general procedure is to collect measured data for the estimation of parameters of quantum systems, which can be regarded as an inversion problem. The emergence of ML offers an alternative automated procedure to capture the characteristics of quantum entities that match the observed data, i.e. to learn a parameterized function by fitting data, which functions as a variational ansatz for quantum systems [80]. One useful choice for the family of parameterized functions can be NNs, which serve as universal function approximators capable of acquiring mappings from noisy input data to output labels. The introduction of NNs enables the average reconstruction fidelity to be improved between 10% and 27% on two-qubit systems compared to a protocol treating SPAM errors by process tomography and a SPAM-agnostic protocol, respectively [30].

Inverse problems deal with determining parameters of interest, $\boldsymbol{w} \in \mathbb {W}$, in a problem involving data $\boldsymbol{f} \in \mathbb {F}$. For quantum estimation problems, quantum measurement involving a set of measurement operators $\boldsymbol{\mathcal {M}}=\lbrace \mathcal {M}_i\rbrace$ maps the quantum entity $\boldsymbol{w}$ to measured frequencies $\boldsymbol{f}$ in a forward way. The goal of quantum estimation is to find the inverse of this process as $\boldsymbol{w} = \mathcal {G}(\boldsymbol{f})$, where $\mathcal {G}$ represents a mapping that transforms $\boldsymbol{f}$ into $\boldsymbol{w}$. Such problems frequently face the challenge of being ill posed, especially when noise becomes a primary contributor and can be amplified during the inversion process. Additionally, the selection of inappropriate measurement operators or insufficient resources for the measurement process can further exacerbate the ill-posed nature of quantum estimation. While altering the measurement operator or acquiring additional data are straightforward solutions to address these issues, they might not be effective in cases where noise amplification during inversion is excessively high. Hence, it is desirable to consider the continuous dependence of the solution on data and the robustness of the model under noise or perturbations.

According to the universal approximation theorem, deep NNs with multiple fully connected layers can act as universal function approximations from $\mathbb {R}^n \rightarrow \mathbb {R}^m$ [81]. Therefore, they can be used to replace the unknown forward and inverse models and extract information from data. For example, one can approximate a map $\boldsymbol{w} = \mathcal {G}_{\xi }(\boldsymbol{f})$ to capture the underlying relationships between $\mathbb {W}$ and $\mathbb {F}$. Function $\mathcal {G}_{\xi }$ is typically a learnable function with parameters $\xi$ to be optimized via minimizing a loss metric that quantifies the disparity between the predictions generated by the NN and the expected labels. The addition of priors to the NN architecture can enable the use of the universal approximation capacity of NNs as well as leverage human knowledge [82]. Striking examples include the design of CNNs from a human visual cortex [12] and the design of Transformers in language models [14]. These approaches have been applied in different quantum estimation tasks on 2–4-qubit systems [30,33,83].

### ML-based quantum state estimation

Compared to traditional methods, NN-based approaches typically involve optimizing a function that fits a large number of data items with minimal errors. A data-driven approach enables the capture of key patterns from data, thus exhibiting robustness against imperfect data. These two features make NNs suitable for state estimation, which involves large-scale measurement data, and imperfections and errors in the measurement process [30,37]. NNs have achieved remarkable success in learning and representing various quantum states, including thermal states [84] and solid-state systems [85]. Here, we focus on QST for finite-dimensional discrete-variable systems, such as multiqubit systems. Many possible architectures of NNs can be employed to characterize quantum states.

#### Restricted Boltzmann machine

**Figure 2.**
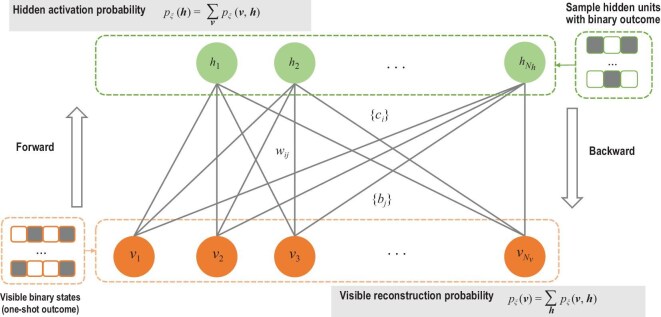
An RBM architecture with a parameter vector $\xi$ (corresponding to an amplitude RBM with $\xi =\lambda$ and a phase RBM with $\xi =\mu$). Each RBM features a set of $N_v$ visible neurons (orange circles) and a set of $N_h$ hidden neurons (green circles) and $\xi$ consists of weights $W$ connecting the layers, and the biases $b$ and $c$ coupled to visible and hidden neurons, respectively. A Gibbs distribution (with normalization omitted) is obtained via $ p_{\xi }(\boldsymbol{v}, \boldsymbol{h})=\exp \lbrace \sum _{i j} W_{i j} h_i v_j+\sum _j b_j v_j+\sum _i c_i h_i\rbrace$ and the distribution over the visible (hidden) layer is obtained by marginalization over the hidden (visible) degrees of freedom [11]. Given visible binary outcomes, the marginal distribution of hidden units is calculated as $p_{\xi }(\boldsymbol{h})=\sum _{\boldsymbol{v}}p_{\xi }(\boldsymbol{v},\boldsymbol{h})$. Based on the sampled hidden configuration, the marginal distribution of visible units is calculated as $ p_{\xi }(\boldsymbol{v})=\sum _{\boldsymbol{h}} p_\xi (\boldsymbol{v}, \boldsymbol{h})$. Two RBMs are trained to minimize the difference between the actual wave function $\psi (\boldsymbol{v})$ and the reconstructed wave function $\psi _{\lambda ,\mu }(\boldsymbol{v})$ (see Equation (15) for detailed information).

A restricted Boltzmann machine (RBM) is an energy-based model that exhibits several parallels with physical systems in statistical mechanics [80,86]. As a variational ansatz, RBMs provide a compact representation for many-body quantum systems, capable of capturing complex correlations, including strong entanglement and topological features. As an example, let us describe spin quantum systems using an RBM (see Fig. 2), which features a visible layer (describing the physical qubits, denoted as a data vector $\boldsymbol{v}=\lbrace v_1,v_2,\dots ,v_k,\dots \rbrace$) and a hidden layer (of binary neurons, denoted as a hidden vector $\boldsymbol{h}=\lbrace h_1,h_2,\dots ,h_k,\dots \rbrace$), fully connected with weighted edges to the visible layer. For QST tasks, each element of the data vector corresponds to the one-shot measurement results, e.g. $\lbrace 1,0\rbrace$. Then, the wave function of a quantum state can be approximated as


(15) \begin{equation*}
\psi _{\lambda ,\mu }(\boldsymbol{v}) = \sqrt{\frac{p_{\lambda }(\boldsymbol{v})}{Z_{\lambda }}} {e}^{\mathrm{i} \log [p_\mu (\boldsymbol{v})] / 2},
\end{equation*}

where $p_\lambda (\boldsymbol{v})$ and $p_{\mu }(\boldsymbol{v})$ represent the approximated amplitude and phase of the state from two RBM networks, and $Z_{\lambda }$ is the normalization constant. The wave function $\psi _{\lambda ,\mu }(\boldsymbol{v})$ acts as a latent model to approximate the wave function $\psi (\boldsymbol{v})$. Note that a complete RBM-based QST approach requires two RBMs, while Fig. 2 provides an illustration of an RBM with a unified parameter vector $\xi$ that consists of the weights connecting the layers, and the biases, and coupled to visible and hidden neurons, respectively. Specifically, $\xi =\lambda$ represents an amplitude RBM and $\xi =\mu$ represents a phase RBM. For QST tasks, $\lbrace \lambda ,\mu \rbrace$ are optimized by minimizing the distance between the reconstructed wave function $\psi _{\lambda ,\mu }(\boldsymbol{v})$ and the real wave function $\psi (\boldsymbol{v})$. One may refer to QuCumber, a Python-supported package designed for quantum state reconstruction using RBM [87], to get more information about the implementation details. Recently, a Python library called QSTToolkit has been developed, which integrates traditional maximum likelihood estimation methods with deep learning–based techniques to reconstruct optical quantum states [88].

The parameters defining the strength of these connections determine the conditional dependencies between neurons, which in turn give rise to intricate correlations among the data variables. Because of their inherently nonlocal nature, the correlations introduced by hidden units are particularly effective in representing many-body quantum systems [80]. This approach has been extended to represent density matrices for mixed states through auxiliary degrees of freedom embedded in the latent space of its hidden units, together with purification [89]. Furthermore, continuous versions of RBMs can be established by replacing the binary encoding (in Fig. 2) with a Gaussian distribution [5]. A distinct advantage of RBMs is their ability to learn directly from raw data, such as experimental snapshots from single measurements. However, this method requires separate training for each new quantum state, as insights gained during the training for one particular state cannot be directly transferred to other states. These issues have stimulated the investigation of more flexible models that can generalize across multiple quantum states.

#### Feedforward networks

Feedforward networks are another class of models to approximate a map function from multiple samples, unlike the RBM that focuses on learning a latent model for one quantum state. Hence, one can build a multilayer network to approximate a function $\boldsymbol{f} \rightarrow \boldsymbol{w}$, where the key is to generate positive semi-definite (PSD) Hermitian matrices from NNs. According to the Cholesky decomposition [90], for any Hermitian definite positive matrix $\rho _H=\rho _H^{\dagger } \ge 0$, there exists a lower triangular matrix $\rho _L$ such that $ \rho _H =\rho _L \rho _L^{\dagger }$. Conversely, given any lower triangular matrices $\rho _L$, one can obtain a density matrix as


(16) \begin{equation*}
\rho = \frac{\rho _L \rho _L^{\dagger }}{\mbox{Tr}\big(\rho _L \rho _L^{\dagger }\big)}.
\end{equation*}

This approach can be extended to generate other quantum entities, for example, POVM elements of quantum measurements [91] and Choi matrices for quantum processes [92–94], as they involve PSD Hermitian matrices. In particular, POVM elements can be obtained by normalizing a set of lower-triangular matrices $\rho _L^k$:


\begin{eqnarray*}
\mathcal {P}_{k} = G^{-1}\rho _L^k \left(\rho _L^k\right)^{\dagger } G^{-1}, \quad G = \sqrt{\sum _k \rho _L^k \left(\rho _L^k\right)^{\dagger }}. \nonumber
\end{eqnarray*}

Similarly, Choi matrices can be generated via


\begin{eqnarray*}
Q_A &=& \sqrt{\mathrm{Tr}_{B} (\rho _L\rho _L^{\dagger})}, \\
Q_{\,\text{Choi}} &=& (Q_{\,A}^{-1}\otimes \mathcal {I}_{B}) (\rho _L \rho _L^{\dagger } Q_{\,A}^{-1} \otimes \mathcal {I}_{B} ).
\end{eqnarray*}

**Figure 3.**
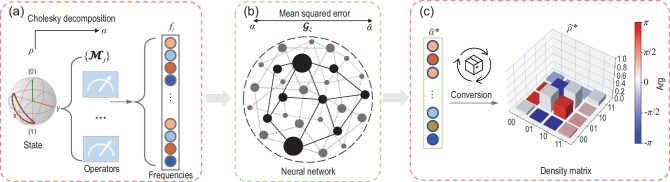
Schematic of NN-based QST. (a) Obtain the measured frequencies $\lbrace f_j\rbrace$ and the ground truth of $\boldsymbol{\alpha }$ via Cholesky decomposition for state $\rho$. (b) A multilayer NN model to map the frequency vector $\boldsymbol{f}=[f_1,\dots ,f_j,\dots ]^\top$ to a predicted real vector $\hat{\boldsymbol{\alpha }}$, and the NN model is trained to minimize the mean squared error between the predicted $\hat{\boldsymbol{\alpha }}$ and the expected value $\boldsymbol{\alpha }$. (c) Obtain a physical density matrix $\hat{\rho }^{*}$ from the optimal $\hat{\boldsymbol{\alpha }}^{*}$. The notation Arg indicates the complex phase (in radians) of each density matrix element.

To deal with real parameters in NN models, the lower-triangular matrices can be further split into real and imaginary parts, ending up with a real vector $\boldsymbol{\alpha }$ [92,93,95]. As demonstrated in Fig. 3, given the frequency vector calculated from the measurement outcomes of different measurement operators, the NN model is required to return a real vector $\hat{\boldsymbol{\alpha }}$ that corresponds to a physical density matrix via Equation (16). The ground truth of vector $\boldsymbol{\alpha }$ is obtained via Cholesky decomposition of the density matrix $\rho$, which has a one-to-one correspondence to $\rho$ [92,93,95], Then, the parameters $\xi$ are trained to minimize the difference between the expected value $\boldsymbol{\alpha }$ and the predicted value $\hat{\boldsymbol{\alpha }}$.

**Figure 4.**
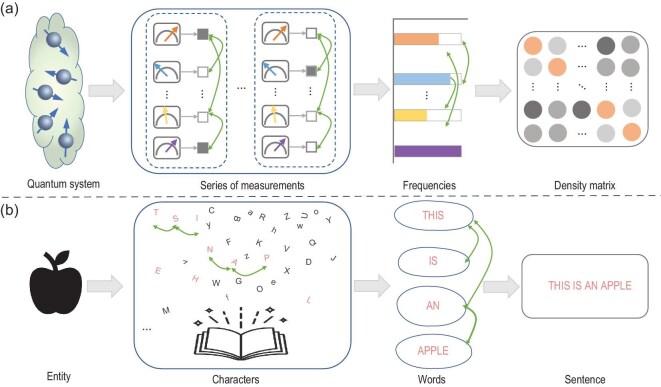
Similarity between QST using structured measurements and the language model using words and characters. (a) Given a quantum system, a series of measurements are performed, each implemented many times, with every one-shot outcome marked as ‘0/1’. Those outcomes are gathered into frequencies that can be utilized to reconstruct the complete state (density matrix) of the involved quantum system. (b) Given an apple, characters are chosen to specify this object, which can be composed into words. Several words then finally specify a full sentence.

NNs are being widely used to characterize quantum states with various architectural designs. Fully connected neural networks (FCNs) were adopted for QST and exhibited potential in the sampling noise due to limited measurement resources [37,96]. By converting measurement outcomes into images [32,83], CNNs have been effective in addressing challenges related to incomplete measurements and adaptive dimensions [35]. Recent attempts have explored sequential information among quantum data [97], focusing on the similarities between quantum patterns and language structure. As demonstrated in Fig. 4, quantum correlations exist at the level of one-shot measurements and the level of expectations of many measurements. Such a hierarchical structure resembles describing an entity using characters, words and, finally, a sentence [95]. In particular, the attention mechanism can be drawn to characterize long-range quantum entanglement among qubits (reflected in projective measurements of the quantum state), benefiting the task of learning the probability function of Greenberger–Horne–Zeilinger states with an order-of-magnitude improvement in the sample complexity compared to RNN-based tomography [36,98]. Another work notes the similarity between words and frequencies of a set of measurements and thus proposes a solution for QST: translating observed frequencies into physical density matrices, thus realizing a full tomography [95], exhibiting an order-of-magnitude improvement in the log of infidelity over FCN methods and CNN methods.

GANs offer a novel approach to learning the mapping between a latent space and data and have been extensively investigated for QST [93,97,99]. In this context, QST is conceptualized as a generative adversarial game involving two players. The generator aims to produce data closely resembling the true data distribution, and the discriminator is trained to distinguish real data from fake data originating from the generator. For QST tasks, it is essential to introduce a variable to control the output, which is known as a conditional GAN. Let the conditional input vector be the composed elements of measured results and measurement operators $\boldsymbol{x}=[\mathcal {M},\boldsymbol{f}]$, and the noise be $\boldsymbol{z}$. The generator functions as a mapping $\lbrace \boldsymbol{x},\boldsymbol{z}\rbrace \rightarrow \rho$. A quantum version of generative adversarial learning has been theoretically proposed to exhibit an exponential advantage over its classical counterpart [100].

Physical quantum systems inherently suffer from limitations, as actual measurement operators and trial states are often imprecisely characterized. Specifically, an NN architecture is constructed to approximate a function mapping the input—measurement frequencies affected by SPAM errors—to the output—ideal, noise-free measurement frequencies [30]. In a two-qubit QST experiment, the network comprises input and output layers with 36 neurons each, and two hidden layers with 400 and 200 neurons, respectively. The NNs are trained on labeled data by minimizing the Kullback–Leibler divergence between pairs of distributions. Once trained, the model can process new data to filter out SPAM-related errors. Quantum state reconstruction is then performed using traditional methods, such as maximum likelihood estimation [30], based on the corrected measurement data. Experimental results show that the NN-assisted approach improves reconstruction fidelity by approximately 10%–27% compared to conventional protocols. RBM-based QST has been utilized to predict properties of two-qubit entangled states from quantum optical experiments [33]. Additionally, quantum generative adversarial networks (QGANs), implemented on superconducting circuit platforms, have successfully learned properties of quantum states, achieving an average fidelity of 98.8% [101]. Quantum extreme learning machines have also been experimentally demonstrated on photonic platforms, enabling resource-efficient and accurate characterization of photon polarization states [102]. Moreover, a range of experimental implementations on the IBM Quantum platform have further highlighted the benefits of ML-based quantum state estimation, particularly in scenarios with limited measurement shots [83,95,103].

### ML-based process estimation

Accurately reconstructing quantum dynamics is essential for tasks like determining channel and gate fidelities in communication and computation, as well as enhancing parameter encoding in quantum sensing [5]. Without imposing any restrictions on quantum dynamics, we first introduce how ML can benefit quantum process tomography. Then we focus on Hamiltonian learning as an illustration. Finally, the dynamics of an open quantum system are investigated.

#### Quantum process tomography

Recall that a quantum process can be defined as a completely positive map $\Lambda$ that transforms an input state $\rho ^{\text{in}}$ to an output state $\Lambda (\rho ^{\text{in}})$ [6]. Quantum process tomography (QPT) refers to the procedure of fully identifying the dynamics governing an unknown quantum system, as in Equation (5). In the standard approach, one estimates the dynamical process by applying it to a set of known quantum states, referred to as probe states $\lbrace \rho _j^{\text{in}}\rbrace$. The output state for each probe state, i.e. $\Lambda (\rho _j^{\text{in}})$, is then reconstructed via QST [104]. To achieve a complete reconstruction of $\Lambda$, the probe states must span a basis for all possible initial states, and the measurements for QST should be tomographically complete. Consequently, full QPT presents greater challenges than QST [105].

According to the Choi–Jamiolkowski isomorphism [61], there exists a one-to-one correspondence between every quantum map $\Lambda$ and a Choi operator $Q_{\,\text{Choi}}$. A normalized $Q_{\,\text{Choi}}$ plays a similar role as a density matrix. This intrinsic analogy between QST and QPT enables all of the theorems about quantum maps to be derived directly from those of quantum states. For example, given $N_P$ probe states and $N_M$ measurements $\lbrace \mathcal {M}_k\rbrace$ for QST, we have the measured frequencies $f_{jk}$ with $j\in \lbrace 1,2,\dots ,N_P\rbrace$ and $k\in \lbrace 1,2,\dots ,N_M\rbrace$. Treating $(\rho _{j}^{\text{in}})^\top \otimes \mathcal {M}_k$ as an entity allows for the conceptualization of a quantum process as a quantum state within a larger Hilbert space. Let the dimension of the system be $d$; then the corresponding dimension of $(\rho _{j}^{\text{in}})^\top \otimes \mathcal {M}_k$ is $d^2$. In principle, QPT can be naturally reduced to QST, for a small number of qubits. Based on these observations, QPT can be simplified as approximating a function that maps $\lbrace f_{jk}\rbrace$ into $Q_{\,\text{Choi}}$. For related information on implementing QPT in Python, one may refer to the QuTiP library [106]. Theoretically, various state estimation techniques can be applied to the characterization of quantum processes as well, including the NN architecture design in the subsection entitled ‘Feedforward networks’.

When prior knowledge about process $\Lambda$ is available, e.g. a unitary process with a fixed number of unknown parameters in the system Hamiltonian, the number of free parameters in $\Lambda$ does not scale as $(2^4n-2^2n)$, but rather as $(2^2n-1)$ for a unitary process with $n$ being the qubit number. In such cases, the estimation can be improved beyond the limitation of exponential scaling of measurement resources, e.g. the number of measurement copies $N_M$. For example, variational algorithms have been employed to reconstruct unitary quantum processes by effectively learning their inverse dynamics [24,107]. Under this framework, a quantum circuit is trained to approximate the action of an unknown unitary by reversing its effect on a known input state. RNNs have been used to model the non-equilibrium dynamics of many-body quantum systems by capturing their nonlinear responses to random external driving [108]. QGAN-based approximations of a quantum map $\Lambda$ have also been proposed to characterize spatially or temporally correlated noise in quantum circuits [99].

#### Hamiltonian learning

In many applications, we may be interested in identifying a unitary process, and only the system Hamiltonian needs to be characterized. For a $d$-dimensional system, the goal is then to identify the corresponding $d\times d$ Hamiltonian matrix $\mathcal {H}$. Various approaches have been proposed for this task, including methods based on the Fourier transform and fitting time-resolved measurements of observables, particularly for few-qubit systems [109]. In some scenarios, partial knowledge of the Hamiltonian’s structure is available, allowing one to assume a parameterized form such as $\mathcal {H} = \sum _m \mu _m X_m$, where $\mu _m$ denotes a set of unknown parameters. Under such assumptions, characterizing many-body Hamiltonians becomes feasible using a polynomial number of parameters. This enables the estimation of the system Hamiltonian even when only a small subset of the subsystems within the quantum network is accessible for measurement. ML, with its strength in extracting patterns from measured data, is particularly well suited for quantum Hamiltonian learning tasks that involve temporal correlations.

**Figure 5.**
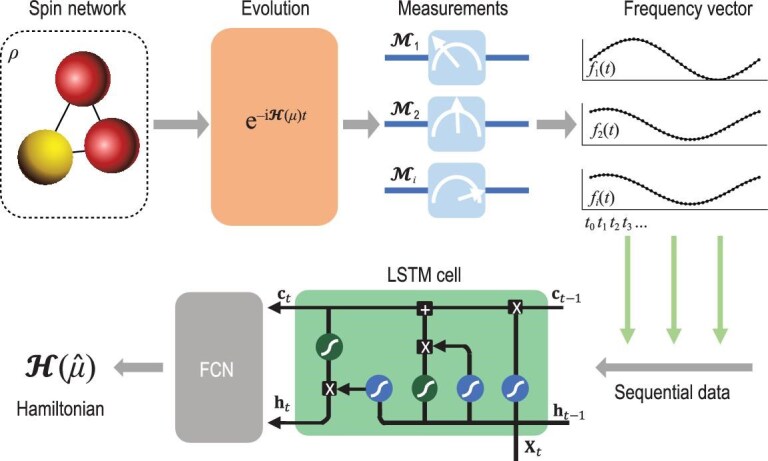
Diagram of NNs for learning the parameters of time-independent Hamiltonians from the temporal records of single-qubit measurements. Starting from the initial state $\rho$, the dynamical evolution $e^{-{\rm {i}}\mathcal {H}(\mu )t}$ is performed, during which the expectation values of single-qubit measurement operators $\lbrace \mathcal {M}_i\rbrace$ (e.g. single-qubit Pauli operators $\sigma _k^{(i)}$ with $k\in \lbrace x,y,z\rbrace$ and $i\in {1,2,\dots }$) are measured at each time step. These expectation values over a period are then collected into a frequency vector $f_i(t)=[f_i^{t_0},f_i^{t_1},\dots ]$, which is fed into an LSTM cell to predict parameters $\hat{\mu }$ of the Hamiltonian.

Prior knowledge of the system Hamiltonian—such as its parameterization using a polynomial number of variables—can reduce the amount of measurement data required for its identification. In such cases, although the relationship between single-qubit measurements and the target Hamiltonian may be complex or governed by unknown functional forms, it can be effectively learned through data-driven machine learning techniques [110]. LSTM (one variant of RNNs) has been employed to learn the Hamiltonians directly from time series data of the single-qubit measurements [110]. As illustrated in Fig. 5, sequential data are injected into an LSTM block, followed by an FCN to reconstruct a time-independent parameter $\hat{\mu }$. For time-independent Hamiltonian learning, one should replace the FCN (gray block) with an additional LSTM module to reconstruct sequential data, i.e. time-dependent parameters $\hat{\mu }(t)$. Strong robustness against measurement noise and decoherence effects has been observed when learning the magnitude and sign of parameters in Hamiltonians, for systems with up to seven qubits.

To overcome the limitations imposed by incomplete prior knowledge of the coupling structure in the original Hamiltonian [110], physics-enhanced Heisenberg NNs have been proposed. These models incorporate a physics-informed loss function derived from the Heisenberg equation, effectively constraining the NNs to respect the quantum evolution of spin variables [111]. Remarkably, even in the extreme scenario where measurements are available from only a single spin, the resulting reconstruction fidelity can reach approximately 90%.

#### Learning open quantum system dynamics

When a quantum system is well isolated from its *environment*, its dynamics can be accurately described by the Hamiltonian formalism discussed above. However, in general, quantum systems interact with their surroundings and should therefore be treated as open quantum systems [60]. For Markovian quantum dynamics, its state evolution can be described by MME (12). Reformulating the evolution equation as $\rho = \mathcal {L}(\rho )$ reveals that the task involves identifying a Liouvillian superoperator $\mathcal {L}$, which operates in a ($d^2 \times d^2$)-dimensional space for a system of dimension $d$. Hence, it is generally more challenging to learn Markovian dynamics than to learn Hamiltonian dynamics.

For non-Markovian quantum dynamics, the state at time $t + \Delta t$, denoted as $\rho _{t+\Delta t}$, depends not only on the current state $\rho _t$, but also on the system’s past evolution. Let $\mathcal {E}_{\le t}$ represent a superoperator that captures the influence of both the current time $t$ and the entire history prior to it. A compact representation for non-Markovian dynamics $\dot{\rho }(t)= \mathcal {L}_{\le t}[\rho ]$ can be obtained as


(17) \begin{eqnarray*}
\dot{\rho }(t) &=& -\mathrm{i}\left[\mathcal {H}+\mathcal {H}_{\le t}^{\rm LS},\rho \right]\nonumber\\
&&+\sum _{\mu } \left [L_{\le t}^{\mu } \rho L_{\le t}^{\mu \dagger }-\frac{1}{2} \left\lbrace L_{\le t}^{\mu } \rho L_{\le t}^{\mu \dagger },\rho \right\rbrace \right ],\nonumber\\
\end{eqnarray*}

where the $L_{\le t}^{\mu }$ are (recurrent) Lindblad operators describing the coupling channel with the environment and $\mathcal {H}^{\rm LS}$ is a ‘Lamb-shift’ term, namely, a correction to the Hamiltonian induced by the environment [112]. This is reasonable because, for small enough $\Delta t$, the time evolution of a quantum state can be simply given as $\rho (t+\Delta t) \approx e^{\Delta t\mathcal {L}_{\le t}[\rho (t)]}$, which is a completely positive trace-preserving quantum map [112]. To capture the long correlations between different time series, RNNs were used to model the long-range memory for non-Markovian dynamics. Directly learning from data also enables us to effectively model complex quantum correlations between systems and environments with a constant and fixed number of parameters. For example, the RNN can be utilized to predict a single recurrent Lindblad $L_{\le t}^{\mu }$ ($\mu =1$) and $\mathcal {H}_{\le t}^{\rm LS}$ (in Equation (17)) for a two-level system with spontaneous decay [112]. CNNs have been employed to predict non-Markovian reduced system dynamics across a wide range of dynamical regimes, spanning from weakly damped coherent motion to incoherent decay [113]. This approach yields small deviations (3.6%) between the predicted and exact populations in two-level quantum systems, while also reducing the computational resources required for long-time simulations.

### Outlook and future directions

ML methods have the potential to achieve improved estimation accuracy in many practical situations, such as few copies and noisy measurement data [32,95]. Some advantages of employing ML methods for quantum estimation include the following: (1) we may not necessarily need complete measurement bases, (2) artificially generated data can be used to train the learner offline for quantum estimation tasks, (3) ML methods can be online integrated into developing adaptive quantum estimation strategies for enhancing estimation accuracy. Still, various challenges deserve further investigation, which opens up new opportunities for future research.

#### Model complexity and scalability.

Additional effort is needed to consider the involved parameters in NN models versus the number of parameters in quantum systems, e.g. ($4^n-1$) free parameters in an $n$-qubit density matrix. Existing achievements in full tomography mainly focus on low-qubit states. As quantum systems grow in complexity, scaling ML algorithms to efficiently process and analyze the increasing amount of data becomes more challenging. Constructing an approximate classical description of a quantum state using very few measurements has been proposed as a classical shadow of quantum states [114]. It would be useful to investigate how to incorporate ML methods to efficiently capture shadows of quantum entities (e.g. quantum channels), therefore predicting the properties of large-scale quantum systems.

#### Benchmarks and accuracy.

Despite the capabilities of ML-based methods in different estimation tasks, it is usually difficult to characterize their accuracy (e.g. fidelity or mean squared error) versus model complexity (parameters in NN models and the resources used), which remains an interesting problem. Although there are some numerical results to determine the scaling of accuracy versus measurement copies of ML-based estimation methods, e.g. Transformer-based QST in [36], it would be useful to obtain an analytic solution to the scaling and whether the scaling could reach the fundamental limit $\sim {1}/{\sqrt{N_M}}$. Considering the additional training overhead in ML-based methods typically absent in traditional methods, it would be interesting to fairly compare the ML-based methods and classical methods with the full consideration of computational complexity.

#### Interpretability.

A significant open challenge is the lack of physical interpretability in black-box approaches, which might be addressed via the integration of prior knowledge from well-established physical laws into the NN architecture. A notable approach is the use of neural ordinary differential equations, which embed the system’s governing differential equations directly into the network structure [115]. Another powerful framework is physics-informed neural networks, where the system’s differential equations are explicitly incorporated into the loss function to guide the learning process [82,116]. Despite these advances, significant challenges remain in developing general ML methodologies that offer strong interpretability when applied to quantum systems.

#### Generalization.

Although ML-based estimation methods demonstrate robustness against different errors, their generalization performance across different types of quantum samples remains inferior to re-training for a new class of samples. This suggests a potential avenue for leveraging relationships between different tasks, thus improving generalization. For example, one to employ advanced ML techniques such as transfer learning to reuse knowledge gained from previous quantum tomography tasks to improve performance on new, but related, tasks. Useful examples might include (1) quantum tomography tasks with varying measurement settings, exploring the relationship between different measurement bases; (2) transitioning knowledge gained from state estimation to closely related tasks such as process estimation by leveraging the similarity between density matrices and Choi matrices, or detector estimation by understanding the relative relationship between the state and measurement. Furthermore, one can also train an *agent* to learn from a distribution of tasks, i.e. meta-learning, to enhance adaptability and transferability across different quantum tasks, e.g. quantum trajectory learning [117], quantum architecture search [118] and quantum gate realization [119].

## LEARNING-BASED OPTIMIZATION FOR QUANTUM CONTROL

Quantum control aims to direct the evolution of quantum systems, with the objective often being to maximize a specific performance function [2]. It can often be formulated as an optimization problem. Learning-based control is an effective approach that can learn from previous experience and optimize system performance by searching for the best control strategy in an iterative way. In the following, we first outline the process of converting quantum control into an optimization problem, highlighting the role of gradient-based methods in addressing this challenge. Following this, we explore the application of evolutionary computing techniques for optimizing quantum systems. We also discuss the experimental applications of learning-based optimization for quantum control. The section concludes with the challenges of learning-based optimization strategies in the realm of quantum control.

### Quantum control as an optimization problem

The objective of quantum control problems can usually be formulated as an optimal control problem. This involves transforming the challenge into the task of optimizing a function, which depends on variables, such as the magnitude of control pulses, and the control time duration [1]. A notion of the quantum control landscape [120] is defined as the map between the time-dependent control Hamiltonian and associated values of the control performance functional $\Phi$, which is usually determined according to practical requirements. For a quantum state preparation task, the fidelity $\Phi =|\langle \psi (T)|\psi _f\rangle |^{2}$ between the target state $|\psi _f\rangle$ and the final state $|\psi (T)\rangle$ or the expectation $\Phi =|\langle \psi (T)| \mathcal {P} |\psi (T)\rangle |^{2}$ of an operator $\mathcal {P}$ may be defined as a performance functional. Define $\langle \mathcal {U}_f|\mathcal {U}(T)\rangle = \mbox{Tr}[ \mathcal {U}_f^{\dagger } \mathcal {U}(T)] /d$. For a quantum gate control problem, the performance functional may be defined as $\Phi =|{\langle \mathcal {U}_f|\mathcal {U}(T)\rangle }|^2$ [121,122].

These problems can be solved using a unified framework of gradient-based methods, where the control fields are iteratively updated in the direction of the gradient of ${\delta \Phi }/{\delta u_{m}(t)}$ with a learning rate $\zeta$. Specifically, for a maximization problem, the control fields can be updated as


(18) \begin{equation*}
u^{k+1}_{m}(t)=u^{k}_{m}(t)+\zeta \frac{\delta \Phi }{\delta u_{m}(t)}.
\end{equation*}

Following this idea, gradient ascent pulse engineering (GRAPE) was developed to maximize the performance $\Phi$ for various quantum control tasks [43]. Another popular method is called the Krotov method [123], where combined information from forward and backward propagation is utilized to update the control fields. This method guarantees monotonic convergence and is well suited for complex and constrained quantum control problems. GRAPE has been extended to open quantum systems based on the quantum master equation [124]. To solve the optimal control problem with constraints in the frequency domain, a gradient-based frequency-domain optimization algorithm has also been developed [125].

In practical applications, robustness is an important requirement due to the existence of uncertainties. Inhomogeneous quantum ensembles, such as collections of atoms, spins or molecules, often exhibit parameter variations across individual systems [44]. These variations may appear as dispersion in radio-frequency field strength or fluctuations in spin resonance frequencies in NMR systems. To apply the same control fields across systems with differing dynamics and drive them from a common initial state to a desired target state, a sampling-based learning control (SLC) method has been proposed [44]. An augmented system consisting of $N_S$ representative samples over the distribution can be constructed, which can be optimized according to the average performance function


(19) \begin{equation*}
\bar{\Phi }(u)=\frac{1}{N_S}\sum _{j=1}^{N_S} \Phi _{\omega ^j}(u),
\end{equation*}

where $\Phi _{\omega ^j}(u)$ represents the objective function for a given sample $\omega ^j$. Applying the SLC idea to GRAPE, a sample-based gradient algorithm (s-GRAPE) has been developed, wherein the control fields can be updated as


(20) \begin{equation*}
u^{k+1}_{m}(t)=u^{k}_{m}(t)+ \zeta \frac{\delta \bar{\Phi }}{\delta u_{m}(t)}.
\end{equation*}

This approach holds promise for inhomogeneous quantum ensembles and quantum robust control [47,121]. Inspired by advanced ML techniques, several gradient-based algorithms have been developed to enhance control robustness while maintaining high fidelity. The batch-based GRAPE, known as b-GRAPE, exploits the richness of sample diversity [126], while the adversarial GRAPE, known as a-GRAPE, improves resilience by generating adversarial samples through the pursuit of Nash equilibria [127]. Additionally, the data-driven GRAPE, known as d-GRAPE, mitigates deterministic gate errors by integrating information from both a design model and experimental data obtained via quantum tomography [128]. Meanwhile, the collaborative GRAPE, known as c-GRAPE, has been developed by combining adaptive QST from the experimental data [129]. According to quantum control landscape theory [120], gradient-based learning methods typically excel in solving optimal control problems when the system model is known and it is usually not trapped in local optimal solutions. However, this assumption may not always hold in experimental setups. To mitigate this challenge, one might resort to an evolutionary computation-based approach to seek effective solutions.

### Evolutionary computation for quantum control

**Figure 6.**
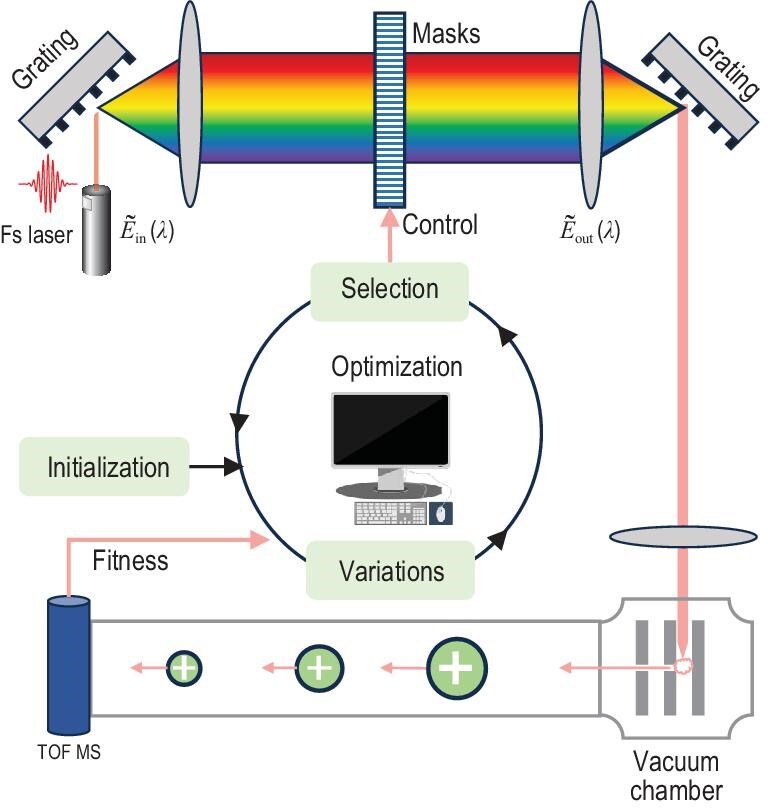
Illustration of the experimental setup of the femtosecond (fs) laser system using adaptive learning algorithms. Laser pulses are directed into a pulse shaper equipped with a programmable dual-mask liquid crystal spatial light modulator, where their phase and/or amplitude are modulated—i.e. the control fields optimized in the inner loop—to shape the pulses. The shaped pulses are then focused into a vacuum chamber, inducing molecular ionization and dissociation, with the resulting charged fragments separated and detected via time-of-flight mass spectrometry (TOF MS). In the inner optimization loop, the variations aim to perform genetic perturbations in the individuals, e.g. ‘mutation’ and ‘crossover’ in GAs and DE [131].

For a quantum control problem, the gradient-based methods typically excel provided that (i) obtaining the gradient is straightforward and (ii) there are no local traps on the control landscape [120]. Nevertheless, ensuring these conditions for complex quantum systems is often challenging. In such cases, leveraging stochastic search algorithms becomes a natural choice for finding effective controls. Here, we delve into evolutionary computation, extensively utilized across various engineering domains, spanning from molecular to astronomical scales [130]. Evolutionary computation algorithms draw inspiration from the natural selection process [130], where the most adept individuals are chosen for reproduction, thereby generating offspring via different variations for the subsequent generation. To implement this concept, it is essential to analogize potential solutions as individuals within a population and to establish a measure of ‘fitness’ based on the quality of the solutions. Consequently, the overall process can be outlined as a loop (see Fig. 6) of evaluating the current generation of solutions, then creating new solutions through different variations and selecting some to act as the basis for the next generation. In the context of genetic algorithms (GAs) and differential evolution (DE), the variation phase mainly comprises two crucial operations: ‘mutation’ and ‘crossover’ [131].

The ‘fitness’ function for each vector corresponds to functional $\Phi (u)$ for each control solution $u$ [132]. Early attempts usually adopted a GA to optimize the ‘fitness’ function of quantum control problems [133]. The GA has also found applications in searching for control pulses for state preparation and quantum gate operations in nuclear magnetic resonance systems [134] and manipulating the ionization pathway of a Rydberg electron [135]. Meanwhile, DE has gained increasing attention in quantum gate control [136]. Despite sharing a similar mechanism, DE has been found to outperform the GA and particle swarm optimization for ‘hard’ quantum control problems [45], such as those requiring short durations for unitary operations or featuring a limited control parameter (for example, low $N_c$ in Equation (10)). An improved DE algorithm introduces an efficient mutation rule that leverages information from both current and previous individuals, which has been validated on quantum state and gate preparation problems on two-qubit NMR systems [137].

Unlike GA methods, which employ binary representation of candidate solutions and a low mutation probability, DE methods represent solutions with real numbers and operate with a higher mutation rate [45]. This enables DE to explore the search space more effectively, diminishing the risk of becoming trapped in local minima, which is particularly crucial for quantum control tasks [46]. Another notable aspect of DE is its versatility in mutation strategy selection since several DE variants based on mixed strategies have exhibited good performance for different optimization tasks [138]. When it comes to the context of quantum control problems, DE with a single strategy may suffice for simple quantum control problems, while DE variants with mixed strategies may be a promising candidate for quantum control problems with multimodal landscapes [46]. To facilitate this, one can construct a strategy pool consisting of several mutation schemes with effective yet diverse characteristics. For example, it has been found that four strategies can yield favorable performance for controlling open quantum systems [46] with high fidelity, with uncertain parameters considered, as well as achieving consensus in quantum networks (all nodes in a network hold the same substates [139]).

In applications where the robustness of the control fields is required, one may either use Hessian matrix information [140] or integrate the concept of SLC into the learning algorithm [139]. Compared with gradient-based methods, DE performs much better when imperfections and measurement errors are involved [141]. Improvements in DE, such as dynamic parameter variation for mutation and crossover [46,139] and the introduction of a direction-adaptive mutation strategy, have resulted in improved robustness and faster processing in handling uncertainties like pulse imperfections and measurement errors [137]. Furthermore, formulating quantum robust control as a multiobjective optimization problem has led to a two-step optimization strategy that prioritizes average fidelity before addressing infidelity variance, thereby bolstering solution robustness [142].

### Adaptive learning control for quantum experiments

When implementing learning methods in experimental quantum systems, the control fields undergo iterative updates to maximize control performance [3]. Since its introduction, GRAPE has demonstrated wide applications in NMR systems, particularly in modules for state preparation [143]. These applications often necessitate computing numerous time propagations of the controlled system’s state, presenting challenges for classical computers, especially in handling high-dimensional systems. To overcome this limitation, researchers have developed methods to approximate the ‘fitness’ function and its gradient for control inputs through evolutionary and measurement processes on a quantum simulator. This approach has facilitated the experimental preparation of complex quantum states, such as seven-correlated quantum states [144]. Experimental verification has been conducted on a solid-state ensemble of coupled electron-nuclear spins [145]. Recently, an iterative GRAPE algorithm has been proposed to decompose large-scale problems into a set of lower-dimensional optimization subproblems through disentanglement operations, with experimental verifications on a four-qubit NMR system [146].

Another groundbreaking advancement is the selective laser modulation of physical and chemical phenomena, enabling the production of numerous samples in identical states for laboratory chemical molecules [2]. Those experiments can be optimized via a closed-loop learning control approach [2], which involves three elements: (1) designing a trial control input, (2) generating and applying this control to a sample in a laboratory setting to observe its effects and (3) employing a learning algorithm that leverages data from previous experiments to update parameter settings to generate new control pulses. As demonstrated in Fig. 6, a setup for the femtosecond pulse-shaping experiment usually consists of a laser, a set of molecules and a measurement device [2]. This closed-loop process is guided by a cost function focused solely on achieving the target molecular state while adhering to experimental constraints (e.g. field limitations). One notable achievement is the use of GAs to control specific molecular transitions [133]. Other achievements include the maximal compression of femtosecond laser pulses [147], e.g. shortening the time width of laser pulses and increasing their peak power for various applications. The adaptive mechanism makes it possible to generate maximally compressed laser pulses simply and effectively, without requiring knowledge of the input pulse’s shape. Meanwhile, DE has also been employed for selective control of molecular fragmentation [139], where $\text{CH}_{2}\text{BrI}$ molecules undergo ionization and dissociation, and their charged products can be separated and detected with time-of-flight mass spectrometry. In this experiment, the control variables are phase parameters, totaling 80 variables. Random noise is introduced with the range of $-7.5\%$ to $+7.5\%$ relative to the maximum phase value of $2\pi$. The control objective was defined as the photoproduct ratio of $\text{CH}_2\text{Br}^{+}/\text{CH}_2\text{I}^{+}$, which corresponds to breaking the weak C-I bond versus the strong C-Br bond. The utilization of DE has contributed good control performance on 100 testing samples. A similar strategy has been employed for controlling the fragmentation of $\text{Pr(hfac)}_3$ using a femtosecond laser [47].

Unlike gradient-based methods, evolutionary computing does not require prior knowledge of the quantum system model, making it well suited for various experimental platforms. Through evolutionary strategies, researchers have determined optimal pulse shapes that significantly enhance the ultrafast semiconductor nonlinearities, nearly quadrupling their effect. This technique has been further applied to coherently control two-photon-induced photocurrents in two distinct types of semiconductor diodes [148]. Researchers designed a learning-based method for quantum experiments by using a Gaussian process model to efficiently optimize the evaporation ramp for Bose–Einstein condensate production, achieving high-quality results with significantly fewer experimental iterations, while also offering insight into the system’s control parameters [149]. Another application is that a genetic algorithm was employed to optimize control pulse waveforms in a warm cesium vapor, achieving excellent performance [150].

### Outlook and future directions

Learning-based optimization of quantum control is a cutting-edge area that focuses on learning techniques to optimize the control of quantum systems. Some advantages of employing a learning-based approach for quantum control include the following: (1) it can be effective without knowledge of the quantum system dynamics; (2) it allows for easy implementations in experimental settings and (3) the adaptive learning approaches bring robustness against possible uncertainties in quantum systems. However, it involves several challenges and opens up various future directions for research and development.

#### Data efficiency.

For each learning trial, a fresh set of quantum ensembles is prepared to obtain the ‘fitness’ function, which can be costly in experimental settings. Population-based methods involve evaluating numerous data points to suggest better solutions, often discarding past individuals and only retaining the current individuals and their associated ‘fitness’. It could be advantageous to store past information in a smart memory, providing insights for future individuals without recalculating ‘fitness’ from scratch. This approach would significantly reduce computational resources and is particularly beneficial for costly experimental implementations.

#### Generalization.

Although control fields discovered through learning-based approaches exhibit robustness against errors in quantum control problems, they are typically tailored for a specific quantum system or task (e.g. a given initial or target state, or a fixed time duration for control pulses). For different problems, the common practice is to start learning from scratch, as the performance of directly applying existing control strategies often degrades. It is highly desirable to consider the similarities between different problems and design control strategies that generalize well across various quantum tasks. This issue, while challenging, is essential for achieving widespread applicability.

#### Real-time implementation.

When applying this approach to experimental devices, additional efforts are required to integrate the learning routine into the entire system, such as using LabVIEW software. This integration can sometimes limit the overall efficiency. Given the popularity and versatility of these methods, it would be useful to design dedicated hardware, such as field-programmable gate array (FPGA) chips, to incorporate these algorithms directly into the devices and improve their sampling capability. This would enhance efficiency and streamline the process.

## REINFORCEMENT LEARNING FOR QUANTUM CONTROL

RL methods offer a considerable advantage in controlling systems without prior knowledge about the *environment* and can be naturally applied to quantum control problems [20]. In particular, RL techniques offer several advantages for quantum control tasks [151]. They can handle complex and high-dimensional quantum systems [70], optimize control policies in real-time [54] and adapt to unknown or changing *environments* [152]. In the following, we first briefly explain how to transform quantum control problems into a decision-making process. Then, we investigate the utilization of RL methods in state-aware quantum tasks. After that, we turn to the case of partial observation, followed by the investigation of quantum error correction. Finally, we outline future directions for RL for quantum control.

### Quantum control as a decision-making process

In the traditional approaches to quantum control, the underlying model of quantum systems is often described using the system’s Hamiltonian or Schrödinger equation [1]. This allows for gradient-based optimization of the cost function [43]. In contrast, model-free approaches do not explicitly model quantum systems, but instead rely on feedback signals from the experimental apparatus [20]. RL approaches offer the distinct advantage of not requiring prior knowledge of complex systems, leading to extensive investigation and applications in various quantum tasks. It is worth noting that in the RL community, methods are generally categorized as model based and model-free. Model-based RL relies on an internal model for predicting future events—often referred to as *planning* [20], which is involved in AlphaGo [21]. In contrast, model-free RL methods optimize *action*-*reward* patterns primarily through trial-and-error learning, circumventing the need for detailed system modeling. This capability has driven significant research into model-free RL in a variety of quantum tasks. In this survey, we focus primarily on model-free RL strategies for optimizing quantum systems, highlighting their flexibility and effectiveness across diverse settings.

**Figure 7.**
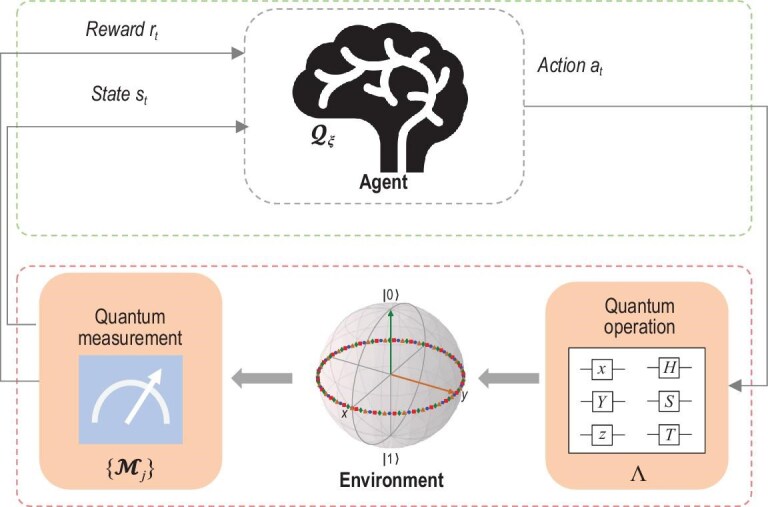
Interaction between an *agent* and *environment* for one step of an RL task for quantum control. The *agent* in the dashed green (classical) part can be represented as suggesting actions based on current states, i.e. $a_t=\mathcal {Q}_{\xi }(s_t)$. The *environment* in the dashed orange (quantum) part is usually a quantum system that is subject to quantum operations for performing *actions*, and quantum measurements for obtaining *reward* signals. Here, a Bloch sphere representation of a qubit is used as an example for illustration.


The process of finding a control policy can be summarized as an *agent* (dashed green part in Fig. 7), aiming to suggest a good action based on the current state, i.e. $a_t=\mathcal {Q}_{\xi }(s_t)$, with $\xi$ representing parameters to be optimized. The control policy can be defined as $a_t=\pi _{\xi }(s_t)$ for policy-based methods or $a_t=\max _{a \in \mathbb {A}} Q_{\xi }(s_t,a)$ for value-based methods. The *environment* refers to the quantum system to be investigated (dashed orange part in Fig. 7). It is important to note the different meanings of the term ‘environment’: in quantum physics, it typically denotes a dissipative bath coupled to the quantum system, whereas in the RL context, it refers to the quantum system itself, which serves as the *environment*. Then, quantum control problems can be generally formulated as a decision-making process: upon observing the current *state*  $s_t\in \mathbb {S}$ (e.g. vector representations of the current quantum state $\rho (t)$, expectation values of some measurement operators or one-shot measurement outcomes of the quantum system), an *action*  $a_t$ (e.g. a set of control $\lbrace u_m(t)\rbrace$ in Equation (10) or a choice from a set of quantum gates) recommended by the RL *agent* is performed on the quantum systems, formulated as a quantum operation $\Lambda$. Based on the (usually unknown) dynamics of quantum systems, the next *state*  $s_{t+1}\in \mathbb {S}$ is obtained, along with a *reward*  $r_t \in \mathbb {R}$ obtained through quantum measurements. Finally, the tuple $(s_t,a_t,r_t,s_{t+1})$ consists of a transition. The training process of RL methods can vary depending on a specific algorithm, but the core principle remains the same: collecting interaction data and iteratively updating the parameters of the *agent* to improve its decision-making policy [20]. As an example, we provide an algorithmic description of DQN for training RL agents for quantum state preparation tasks; detailed steps can be found in Algorithm 1.

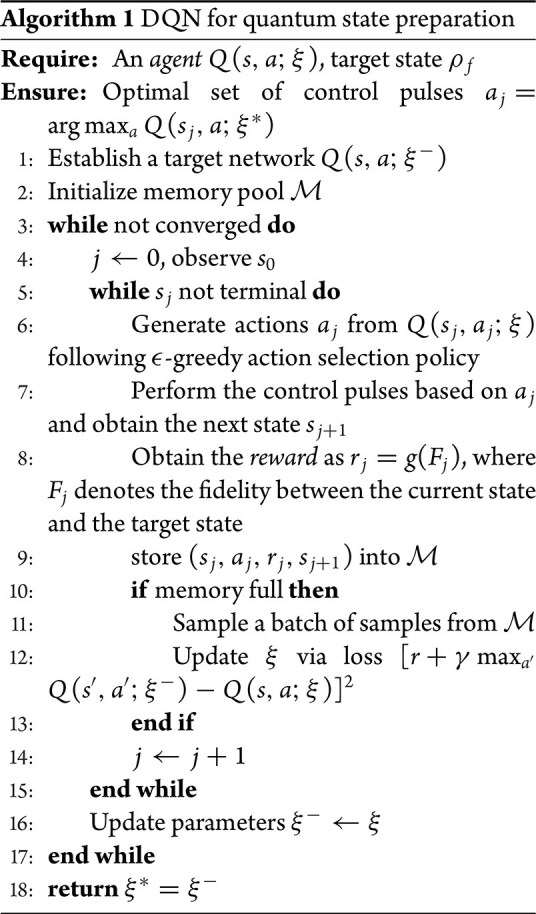

Remark 1. *In deep RL, theoretical convergence remains an open question. DQN uses techniques like experience replay and target networks to improve stability, achieving practical convergence under certain assumptions (e.g. smooth function approximation, bounded updates and sufficient exploration). However, a formal proof of convergence in general settings remains a challenge. Policy gradient methods, such as proximal policy optimization (PPO) and trust region policy optimization (TRPO), employ stability-enhancing techniques and show empirical success, but their guarantees are typically limited to local convergence due to the non-convexity of deep NNs.*

It is worth highlighting that the design of appropriate *reward* functions plays a crucial role in guiding an RL *agent* towards effective policies [52]. In quantum control tasks, one can set *reward* signals based on fidelity information $\mathcal {F}_t$, with non-linear transformation functions, e.g. $r_t=-\log _{10}(1-\mathcal {F}_t)$ [52,153] or $r_t=\mathcal {F}_t^j$ [154], where $j$ could be a positive integer to control the non-linear transformation. By iteratively exploring the *environment* with the past transitions collected, parameters $\xi$ are updated to maximize the cumulative *reward* among a sequence of transitions $\sum _{t =0} \gamma ^t r_{t+1}$ [20]. As such, the *agent*  $\mathcal {Q}_{\xi }$ can discover optimal control strategies that lead to high performance (e.g. high fidelity) for quantum systems [50]. In practical scenarios, the observations from quantum systems usually involve high-dimensional problems, creating a strong need for deep RL methods, such as TRPO [72] and PPO [73] to tackle quantum tasks [48,154–159].

In quantum contexts, the observation of a quantum system would be described by a measurement mapping in a state space model that depends on the current state and the previously applied action. Those situations fall into the paradigm of partially observable MDPs, where the observation is dependent on the current *state* and the previous *action* [67]. In particular, the expectation values of a projector $\mathcal {P}_i$, i.e. $p_i=\rm {Tr}(\rho \mathcal {P}_i)$, or even the one-shot measurement outcome of $\mathcal {P}_i$, i.e. $\lbrace 0,1\rbrace$, represent the partial *state* of the *agent*. Taking into account the specific characteristics of quantum systems, such as the challenge of obtaining a full description of quantum states, application of RL to quantum systems can be divided into two areas: (1) *state-aware* quantum tasks where full observability of quantum systems are available, in which $s_i$ can be obtained from $|\psi \rangle$ or $\rho$, e.g. via splitting imaginary and real parts or other transformations (one should note that the acquisition of full knowledge about quantum states is reasonable when system models and initial states of quantum systems are known or informationally complete measurements on a large scale of identical copies are available for QST); (2) quantum tasks with *partial observability*, where only measurement expectations or one-shot measurement outcomes of measurement operators are available.

### RL for state-aware quantum tasks

RL techniques offer several advantages for quantum control tasks, including their ability to manage complex and high-dimensional quantum systems [158], optimize control policies in real time [48] and adapt to unknown or dynamic *environments* (i.e. quantum systems) [151]. In the early stages, $Q$-learning was utilized to identify variational protocols with nearly optimal fidelity [153], even in challenging situations, such as the glassy phase [160] and quantum optics experiments [161]. In recent decades, various proposals have emerged for applying DRL to a wide range of quantum control problems. These include quantum state preparation [51,153,155], quantum gate construction [50,158,162], quantum metrology [57–59,163], quantum simulation [164], quantum spin squeezing [159] and the quantum approximate optimization algorithm (QAOA) [53]. Here we use two classes of quantum control problems to demonstrate the applications of RL: (i) coherent quantum control and (ii) measurement-based feedback quantum control. We refer the reader to Table 2 for the specific applications of RL methods.

**Table 2.** Different RL methods for quantum control applications.

Quantum applications	Control level	Adopted RL methods
Quantum state preparation	Hamiltonian control	DQN [52,170,171]
		Policy gradient [171]
		PPO [155]
Quantum gate control	Hamiltonian control	DQN [162]
		Policy gradient [172]
Extreme spin squeezing	Hamiltonian control	PPO [159]
Quantum metrology	Hamiltonian control	A3C [57]
		DDPG [163]
Quantum compiler	Gate control	DQN [55,167]
Quantum state engineering	Measurement based	DQN [170]
Quantum state stabilization	Measurement based	DQN [169]
		PPO[54]
Quantum error correction	Measurement based	Policy gradient [51]
		PPO [48,54]

#### Coherent quantum control (Hamiltonian control)

Let us consider a quantum system with Hamiltonian $\mathcal {H}(t)=\mathcal {H}_0+\sum _m u_m(t) \mathcal {H}_m$. The goal of RL is to discover a set of sequential control pulses $u(t)=\lbrace u_m(t)\rbrace _{m=1}^{N_c}$ to drive the quantum system to yield optimal performance. Piecewise control fields are widely used where the control is fixed during a short duration $\Delta t$. In this regard, for each time step $j$, we consider the system Hamiltonian to be constant in $[j\Delta t,(j+1)\Delta t]$. According to Equation (10), the coherent quantum control for time duration $\Delta t$ amounts to a unitary transformation


\begin{equation*}
\mathcal {U}_{j}=\exp \bigg [-{\rm {i}}\Delta t\bigg (\mathcal {H}_0+\sum _m u_m (j\Delta t)\mathcal {H}_m\bigg )\bigg ].
\end{equation*}

At each time step $j$ with the current *state*  $s_j$ (from $\rho (j\Delta t)$ or $|\psi (j\Delta t)\rangle$), the RL *agent* suggests an *action*  $a_j$ (corresponding to $u_n(t)$) that contributes to a system Hamiltonian $\mathcal {H}(j\Delta t )=\mathcal {H}_0+\sum _m u_m(j\Delta t)\mathcal {H}_m$. Then the quantum system evolves into the next state according to the unitary transformation $\mathcal {U}_j$ associated with the current Hamiltonian $\mathcal {H}( j \Delta t )$. Possible observation of quantum systems yields some *reward* signals $r_j$, e.g. fidelity. Meanwhile, the transition $(s_j,a_j,s_{j+1},r_{j})$ is collected for updating the parameters in the RL *agent* [20].

This proposal has been extensively explored in different tasks. For example, DRL approaches have been employed to discover optimal driving protocols for global state preparation within the two-dimensional subspace represented by the Bloch sphere [155]. Interestingly, these protocols tend to cluster into groups with shared characteristics, offering insights into underlying physical constraints. The TRPO algorithm has been used to simultaneously optimize both the speed and fidelity of quantum gates, while addressing leakage and stochastic control errors [50]. The potential of DRL has also been demonstrated in faster state preparation across a quantum phase transition [157]. Enhanced DRL techniques further improve performance in specific systems, including achieving faster transfer than standard Gaussian pulses in semiconductor quantum dot arrays [165], and enabling precise manipulation of Ag adatoms on Ag(111) surfaces, with success rates reaching 95% after adaptation to new tip conditions [166].

When focusing on a sequence of quantum gates $\mathcal {U}_1, \mathcal {U}_2,\dots , \mathcal {U}_j,\dots$ rather than a sequence of control pulses, the problem of finding control pulses that achieves a desired transformation can be simplified. It involves decomposing one gate into a sequence of elementary gates, i.e. a finite universal set [56]. Given several elementary gates (e.g. $H$, $S$, $T$, controlled-NOT gates, etc.), the *agent* aims to select from the above candidate pool to realize a quantum gate capable of performing a desired task. For example, an arbitrary single-qubit gate can be compiled into a sequence of elementary gates from a finite universal set [167]. An RL-based quantum compiler has been developed to realize two-qubit operator compiling [55].

#### Measurement-based feedback quantum control

In quantum feedback control, measurements on a quantum system generally perturb the system’s state, introducing measurement-induced noisy dynamics, commonly known as quantum backaction [6,168]. Recent efforts have been made to evaluate the performance of optimized feedback or adaptive measurement protocols using RL techniques. For example, RL has shown its capacity to effectively learn counterintuitive strategies for cooling a double-well system to a state closely resembling a ‘cat’ state, exhibiting high fidelity with the true ground state [169]. State-of-the-art DRL techniques have enabled the implementation of measurement-based feedback control to generate and stabilize Fock states in a cavity under quantum non-demolition photon-number detection [154]. Compared to traditional methods that rely on control Lyapunov functions for state stabilization, the DRL-based method works well without prior knowledge of quantum models.

### RL for quantum control with partial observation

For quantum control with partial observations, quantum measurements (e.g. projective measurements) are utilized to capture limited knowledge about the system throughout the process. These intensive measurements raise several challenges. (i) Partial observations of quantum states, such as statistic-measured frequencies for corresponding measurements [172] or quantum properties (e.g. coherence, entanglement), can be collected and represented as the partial *state* fed into the RL *agent*. The reduced information in the partial *state* representation might hinder the performance of learning by trial and error. (ii) The measurement process causes a random discontinuous jump in the underlying state [6]. In RL, partially observable dynamics are typically represented by partially observable Markov decision processes (POMDPs). In the context of quantum systems, the inherently partial observability has motivated the study of quantum-observable Markov decision processes (QOMDPs)[173].

Despite the inherent challenge of partial observability in quantum systems [51], RL has proven itself to be a versatile tool to learn directly from stochastic measurement outcomes or low-sample estimators of physical observables. For example, equipped with expectation values of the adjacent pairs of a 128-spin Ising chain, an RL *agent* has successfully devised policies converging to the optimal adiabatic solution for a QAOA [156]. Given only an incomplete Bloch vector representation (i.e. expectations of partial elements among the generalized complete Pauli operators, ${\rm Tr}(\rho \sigma _k),\, k \in \lbrace x,y,z\rbrace$), RL methods have attempted to realize high-fidelity quantum state preparation and QAOA applications [53]. Furthermore, an RL *agent* has been designed to learn from the estimated density matrices based on measurement outcomes via QST, enabling the experimental realization of single-qubit gates on a superconducting quantum computer without prior knowledge of a specific Hamiltonian model, control parameters or underlying error processes [172]. Researchers have taken a step further to explore the utilization of one-shot measurements rather than statistical values. For example, a *state-aware* model using simulated data assists in the effective training of a *state-unaware* model that suggests control based on experimental data of one-shot measurement outcomes [51]. Recently, efforts have brought RL closer to quantum observability, introducing an RL framework that relies exclusively on measurement outcomes as the sole source of information about the quantum state [54]. In these settings, the *state* representation may have a variable length, considering the temporal structure of the control sequences. Then, it is beneficial to utilize advanced architectures like LSTM [54] and Transformer [14]. It is promising to investigate the integration of the QOMDP framework [173] to extract quantum features from data. Such an approach can systematically address the inherent partial observability in quantum systems and potentially yield significant performance gains in the manipulation of quantum systems.

The advantage of RL in handling quantum systems with partial observation has made it a promising tool for experimentally controlling such systems. In early applications to quantum systems, RL was experimentally deployed, but the training was primarily conducted in simulation [157,174]. A common strategy involves pre-training the model using guidance from traditional methods—for example, by designing a reward signal based on shortcuts to adiabaticity that penalizes deviations from linear detuning growth—and then fine-tuning it using an alternative reward under random systematic errors [175]. Subsequent pioneering works demonstrated fully experimental training of RL agents, including the acceleration of the tuning of quantum dot devices [176] and direct optimization of quantum gate pulses for single-qubit gates on a superconducting quantum processor [172]. Recently, an enhanced RL approach was experimentally trained using only measurement data to initialize a qubit with real-time feedback in superconducting systems [49]. To allow for real-time feedback on quantum devices, a low-latency NN architecture was specifically implemented on FPGAs to process data concurrently with data acquisition. In this experiment, the *state* is represented by time traces of the two quadrature components of the digitized signal, while the *action* is selected from three options: *Flip, Idle* or *Terminate*. The *reward* signal is determined by the integrated outcome of the final verification measurement, which reflects the ground-state population. Trained after 30 000 episodes using a policy gradient method, the quantum state initialization error converges to approximately 0.2%.

Given the inherent stochasticity in quantum mechanics [6], POMDPs for quantum control often favor stochastic policies over deterministic policies [54]. Unlike a deterministic policy, a stochastic policy capable of generating probabilistic *action* may compensate for the randomness inherent in the quantum measurement process. Algorithms like PPO that perform small updates within the trust region have found wide applications in various quantum tasks [48,54,154,155,157–159]. In experimental settings, there is a need to reduce the sampling time for obtaining *reward* signals. One approach was proposed to define a reduced distance metric based on partial state representations at each step, e.g. the distance between the actual partial Bloch vector representation and the target partial Bloch vector representation [53]. To take it further, one may provide the *reward* signal at the end of the episode and use the *reward*  $r_{t<T}=0$ at all intermediate time steps [48,54,158,170]. Considering that a *reward* signal generally involves a measurement process that inevitably disrupts the quantum state, reducing the sampling of *reward* signals mitigates the impact of state collapse (random jumps). While this strategy does offer the advantage of high experimental sample efficiency [54], it can potentially disrupt the guiding learning process, which traditionally relies on accurate *reward* signals. To address the sparse *reward* signal in partial observation settings, one can introduce an auxiliary task to predict *reward* signals [177].

### RL for quantum error correction

Quantum error correction (QEC) stands out as a cornerstone, being widely recognized as the crucial basis for achieving fault-tolerant quantum computation. Among the various QEC strategies, stabilizer-code-based QEC represents a widely adopted framework, which systematically involves four key steps: encoding, error detection, error correction and decoding [178]. Surface code has emerged as one of the most experimentally feasible QEC schemes due to its threshold properties and local stabilizer measurements [178]. Recent advancements by Google have demonstrated quantum error correction below the surface code threshold, marking a significant step towards scalable fault-tolerant quantum computation [179].

**Figure 8.**
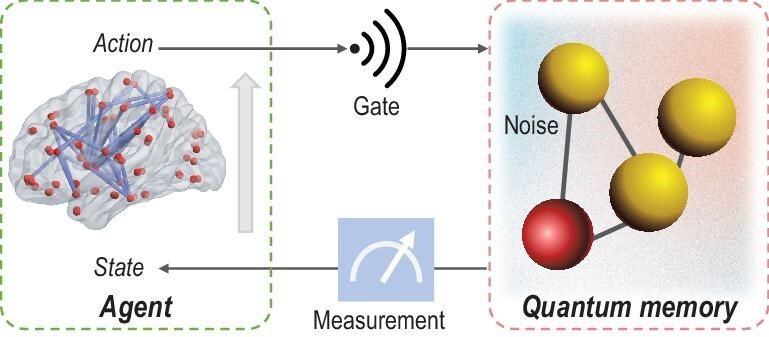
Schematic of protecting quantum memories (qubit network) from the detrimental effects of noise via RL. Given a quantum device consisting of a few qubits, the RL *agent* is required to take *actions* (i.e. making a selection from gate sequences, and the execution of measurements). To obtain the optimal effects, the RL *agent* responds to measurement outcomes and collects *reward* signals to guide the RL *agent* towards good *actions*.

A complete QEC protocol necessitates the cooperation of multiple quantum and classical components, including quantum hardware, real-time error syndrome extraction and classical decoding algorithms. In this context, RL helps guide the *agent* to perform certain *actions* in the form of fault-tolerant local deformations of the code, thus benefiting the processes of detection and correction. Figure 8 illustrates an example of applying RL to discover an adaptive strategy that protects quantum memory against noise [51]. In this context, the *environment* comprises a quantum memory affected by noise and a classical control system responsible for executing QEC. During each interaction cycle, the *agent* receives observation input from the *environment*, reflecting the current status of the quantum device. The objective of RL is to identify an optimal sequence of *actions*—such as quantum gates and quantum measurements—that enables the *agent* to respond effectively to changes in the quantum memory. To this end, the *agent* receives rewards when the error rate falls below a predefined threshold, indicating a successful protection.

Remarkable achievements have been realized in the field of RL for QEC [48,51,54,180]. A notable achievement is the development of a unified, fully autonomous framework using policy gradient methods to discover QEC strategies from scratch in few-qubit systems under arbitrary noise and hardware constraints [51]. The success in preparing stabilizer states in an oscillator further demonstrated the capability of DRL methods [54]. Building on this, a fully stabilized and error-corrected logical qubit has been realized, exhibiting quantum coherence times significantly exceeding those of all imperfect components involved in the QEC cycle [48]. In this implementation, QEC circuit parameters are trained *in situ* using PPO, enabling the system to adapt to actual error channels and control imperfections.

While QEC can be addressed using RL, it is crucial to take into account (i) measurement-based feedback: apart from typical quantum gates, projective measurements can be included as possible *actions* that manipulate dynamics of quantum states [51,54]; those measurement-based *actions* may introduce discontinuous jumps of quantum states, introducing intricate physical dynamics of quantum systems; and (ii) both recording and correcting errors typical during a complete QEC. It is desirable to design different *reward* signals to distinguish different QEC levels [51]. One may define a ‘protective’ *reward* signal to keep the large quantum recovery information among the quantum states [51]. If one wishes to fully decode the quantum state, a ‘recovery’ *reward* that considers the overlap between the original state and the recovered state may be designed to encourage operations that can correct errors of quantum states.

### Outlook and future directions

RL provides a promising framework for tackling the challenges of quantum control, enabling the development of effective and adaptive control strategies in quantum systems. Compared to the learning-based approaches in the section entitled ‘Learning-based optimization for quantum control’, RL methods exhibit the following notable advantages. (1) The introduction of a *reward* signal at each step throughout the entire control pulse, rather than a single ‘fitness’ value after the control pulse, allows for flexible control of the quantum system (e.g. varied control pulses). (2) Incorporating NNs in RL enables effective optimization of quantum systems under challenging conditions, such as partial observation of the quantum systems available. (3) DRL methods aim to learn *state*-*action* patterns from large-scale data (i.e. the previous experience via trial and error), demonstrating improved robustness against errors. Despite these advantages, several open challenges remain, and RL for quantum control deserves further development.

#### Open quantum systems.

A quantum system inevitably interacts with an external system, commonly referred to as the environment or bath. This interaction modifies the system’s dynamics, leading to quantum dissipation, which increases the difficulty of controlling quantum systems towards desired states. In quantum feedback control, imperfections in the measurement process also constrain the amount of information that can be extracted from the system, thereby degrading control effectiveness. Although there are some attempts to apply RL to control open quantum systems [52,154,158,169,181], achieving good control in the presence of various imperfections, noise or even non-Markovian dynamics remains a significant and open challenge. It deserves further investigation to characterize different patterns in open quantum systems using advanced ML techniques to facilitate an improved control performance regarding different imperfections.

#### Sample efficiency.

The control performance of complex quantum systems may be hindered by sparse *rewards*, as early transitions may not achieve a good fidelity, thus providing little information to train the RL *agent*. Without additional measures, it may require a large number of training iterations to find an effective control. Utilizing *reward*-shaping techniques that learn *reward* signals from NNs, rather than relying on predefined human-designed *rewards*, may encourage more effective exploration, thus improving sample efficiency. Additionally, incorporating advanced learning strategies such as curriculum learning or transfer learning may enable efficient exploration of quantum systems by reusing knowledge gained from existing experiences. This approach reduces the need to train on a large number of new samples to capture useful *state-action* patterns, thereby enhancing overall efficiency.

#### Real-time implementation.

A key challenge in applying RL to quantum experiments is the disparity between the classical processing timescale and that of quantum systems. To implement control suggested by the RL *agent* on a submicrosecond timescale, it is crucial to design a low-latency NN architecture allowing for processing data concurrently with data acquisition on hardware, such as FPGAs. The reduced latencies in the data processing and analysis in the *agent* are key to real-time control of quantum systems.

## CONCLUDING REMARKS

In this review, we have investigated various quantum tasks, considering the complexity of quantum systems, as well as the intrinsic probabilistic nature of quantum measurements. Notably, ML techniques are frequently applied to capture information about quantum systems via post-processing routines or to manipulate them toward desired targets. The advantages of NNs for quantum estimation, learning-based optimization of quantum systems and RL for quantum systems highlight the significant power of ML in addressing quantum challenges.

One limitation of ML-based approaches is their inherent black-box nature, which hinders interpretability and trust in the learned models. Recently, physics-informed ML methods have emerged as a promising direction, allowing models to incorporate underlying quantum principles during the learning process, thereby enhancing interpretability [82,116]. Meanwhile, improving the generalization capabilities of ML in quantum settings has become a critical challenge, leading to the exploration of meta-learning techniques that leverage prior knowledge from similar quantum systems to accelerate adaptation and improve performance [117,118]. Quantum ML is promising in exploiting quantum-specific patterns, ultimately enabling more accurate estimation of quantum systems, and efficient control of quantum systems [28,29]. These promising directions will drive advancements across the field.
